# Evaluation of Groundwater Potential Zones Using GIS‐Based Machine Learning Ensemble Models in the Gidabo Watershed, Ethiopia

**DOI:** 10.1002/gch2.202400137

**Published:** 2024-10-16

**Authors:** Mussa Muhaba Mussa, Tarun Kumar Lohani, Abunu Atlabachew Eshete

**Affiliations:** ^1^ Faculty of Water Resources and Irrigation Engineering Water Technology Institute Arba Minch University Arba Minch P.O. Box 21 Ethiopia; ^2^ Faculty of Hydraulic and Water Resources Engineering Water Technology Institute Arba Minch University Arba Minch P.O. Box 21 Ethiopia

**Keywords:** geographic information systems, gidabo watershed, groundwater potential zones, random forest, support vector machine

## Abstract

The main objective of this study is to map and evaluate groundwater potential zones (GWPZs) using advanced ensemble machine learning (ML) models, notably Random Forest (RF) and Support Vector Machine (SVM). GWPZs are identified by considering essential factors such as geology, drainage density, slope, land use/land cover (LULC), rainfall, soil, and lineament density. This is combined with datasets used for training and validating the RF and SVM models, which consisted of 75 potential sites (boreholes and springs), 22 non‐potential sites (bare lands and settlement areas), and 20 potential sites (water bodies). Each dataset is randomly partitioned into two sets: training (70%) and validation (30%). The model's performance is evaluated using the area under the receiver operating characteristic curve (AUC‐ROC). The AUC of the RF model is 0.91, compared to 0.88 for the SVM model. Both models classified GWPZs effectively, but the RF model performed slightly better. The classified GWPZ map shows that high GWPZs are typically located within water bodies, natural springs, low‐lying regions, and forested areas. In contrast, low GWPZs are primarily found in shrubland and grassland areas. This study is vital for decision‐makers as it promotes sustainable groundwater use and ensures water security in the studied area.

## Graphical abstract

This study uses GIS‐based Random Forest (RF) and Support Vector Machine (SVM) machine learning models to evaluate groundwater potential zones (GWPZs) in the Gidabo watershed utilizing data from 75 boreholes and springs. Water bodies, springs, low‐lying areas, and forests all have good GWPZs, while shrubland and grassland have poor GWPZs.

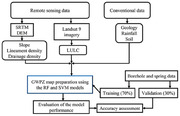

## 1 Introduction

Groundwater is the water that lies below the surface of the Earth, in the saturated zone, beneath the water table, and within soil and rock formations. It is replenished by precipitation via infiltration and percolation. Estimating the global groundwater is difficult due to considerable variances among different sources. Groundwater represents about 30% of the freshwater, while the rest is divided among wetlands, lakes, rivers, the atmosphere, sea ice, glaciers, polar ice caps, and permanent snow.^[^ ^1^ ^]^ Groundwater is the most extensively utilized freshwater source worldwide, meeting various needs such as drinking (50%), and irrigation (40%), and the rest is utilized for industrial and miscellaneous needs.^[^ ^2^ ^]^ Groundwater resources have been depleted due to climate change, industrialization, rapid urbanization, and intensified agriculture.^[^ ^3^ ^]^ Thus, evaluating groundwater potential zones (GWPZs) helps communities and policymakers pinpoint areas with high potential for sustained extraction and utilization and regions where groundwater resources are limited or in danger of depletion.

Most towns and communities in Ethiopia rely on groundwater sources like improved cold springs, boreholes, and hand‐dug wells for domestic water needs. Groundwater plays a crucial role across Ethiopia's different climatic regions, providing over 80% of the water used for domestic needs by urban and rural populations.^[^ ^4^ ^]^ Rain‐fed farming is the dominant agricultural system in all climatic regions of Ethiopia, and crop production is hampered by rainfall variability.^[^ ^5^ ^]^ [Supplementary-material gch21649-supitem-0001]

Communities in the Gidabo watershed experience limited domestic water supply, particularly during the dry season.^[^ ^6^ ^]^ Agriculture is the main land use in this watershed, which relies solely on rainfall and is thus vulnerable to seasonal variations. Furthermore, the region is experiencing rapid population growth and urban expansion, putting further strain on the already scarce water resources.^[^ ^7^ ^]^ Despite these challenges, the GWPZs in the Gidabo watershed have yet to be investigated. This suggests a significant research gap, as evaluating GWPZs is vital for long‐term water resource management in the region. Evaluating GWPZs can provide insights into the most suitable areas for groundwater extraction and maintaining a consistent water supply for domestic and agricultural purposes. Mapping these zones also helps to establish resilient farming practices, mitigate the adverse impacts of rainfall variability, and improve the efficient utilization and distribution of water resources. Addressing this gap would improve water security and enhance socio‐economic development in the Gidabo watershed and its surrounding communities.

Both conventional and advanced methods are used to locate potential groundwater sites. Geophysical, sample drilling, and pumping tests are conventional methods for identifying and evaluating prospective groundwater sites.^[^ ^8^ ^]^ Conventional methods for locating potential groundwater areas rely heavily on ground surveys, however, more advanced techniques use remote sensing (RS) and geographic information systems (GIS) coupled with multi‐criteria decision‐making (MCDM) techniques and machine learning (ML) algorithms. Conventional procedures are laborious and expensive, but GIS and RS techniques are fast and inexpensive.^[^ ^9^ ^]^

Many researchers have used combined MCDM variants (Fuzzy‐Analytical Hierarchy Process (Fuzzy‐AHP),^[^ ^10^ ^]^ Multi Influencing Factor (MIF),^[^ ^11^ ^]^ and AHP^[^ ^12^ ^]^) with RS and GIS to locate prospective groundwater areas. However, MCDM techniques such as AHP's reliance on subjective inputs and constraints in dealing with large and complex datasets can limit its use.^[^ ^13^ ^]^ In contrast, ML excels at processing massive amounts of data and employing algorithms to identify intricate patterns, boosting the precision and reliability of prospective groundwater zones. When integrated with RS and GIS for spatial data analysis, ML enhances the accuracy of identifying probable groundwater zones.^[^ ^8^ ^]^ The latest ML models utilized for mapping GWPZs include random forest (RF),^[^ ^14^, ^15^, ^16^ ^]^ decision trees,^[^ ^17^ ^]^ support vector machine (SVM),^[^ ^18^ ^]^ and convolutional neural networks,^[^ ^19^ ^]^ which incorporate many factors influencing groundwater occurrences and flow.

With recent advancements in data science, RS, and GIS, researchers increasingly use GIS‐based ML techniques like RF and SVM to map GWPZs.^[^ ^20^, ^21^ ^]^ The GIS‐based RF model produces more reliable and precise results by reducing overfitting and underfitting, two main concerns with ML, and effectively handling high‐dimensional data. As a result, it is gaining popularity.^[^ ^22^, ^23^ ^]^ This study employed GIS‐based ML models like RF and SVM to locate GWPZs in the Gidabo watershed.

This research aims to map GWPZs in the Gidabo watershed using GIS‐based ML models. The specific objectives are to: 1) prepare thematic layers influencing groundwater occurrence, 2) train RF and SVM models with data from springs and boreholes, 3) generate a GWPZ map with the models used, and 4) evaluate the performance of these models. Seven factors (drainage density, geology, lineament density, rainfall, soil type, land use/land cover (LULC), and slope) were included, as they influence the flow and distribution of groundwater. While ML models are commonly employed for GWPZ mapping in other countries, their use in Ethiopia, notably in the Gidabo watershed, is limited. This study is vital since the area experiences water scarcity during the dry season. To address this issue, two advanced ML models (RF and SVM) were employed to map GWPZs. The performance of these models was evaluated using a confusion matrix, alongside metrics such as the Kappa coefficient, overall accuracy, and Area Under the Curve (AUC). Its novelty lies in applying and evaluating two ML methods in an underexplored area, assessing their practicability and performance. This approach offers useful insights for groundwater professionals, decision‐makers, and government agencies aiming to plan water resources sustainably.

## 2 Experimental Section

### 2.1 Study Area

**Figure 1:**
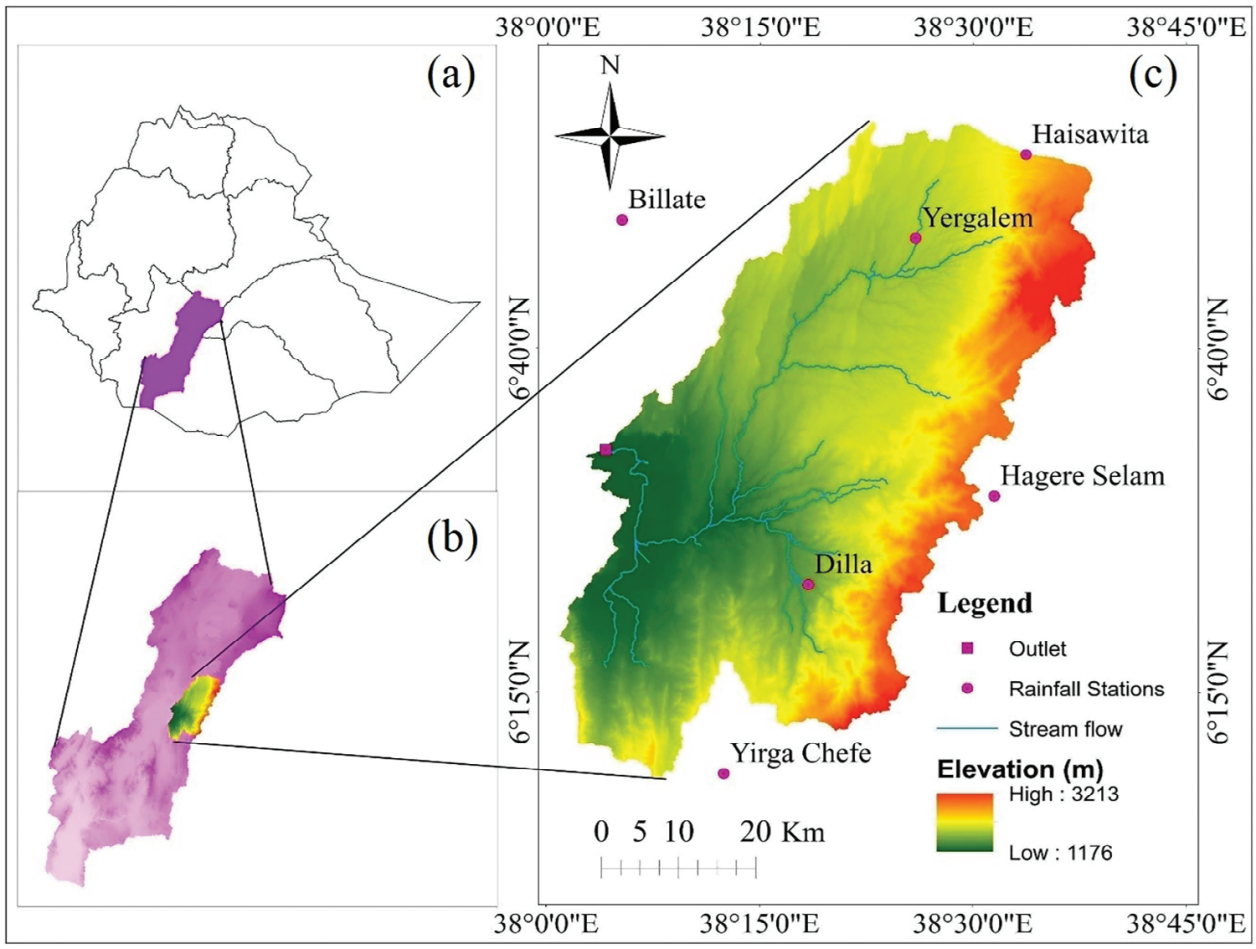
Location map of the study area: a) Ethiopia River Basins, b) Rift Valley Lakes Basin, c) Gidabo watershed.

The Gidabo watershed was positioned in Ethiopia's Rift Valley Lakes Basin, between the latitudes 6° 9′ 25′' and 6° 56′ 13′' N and the longitudes 38° 0′ 50′' and 38° 38′ 24′' E. It had a total area of ≈3297.74 km^2^ and an elevation varying from 1176 to 3213 m above mean sea level (**Figure** **1**). The predominant physiographic featured within this watershed include undulating plains, steep riverbanks, valleys, and cliffs. This watershed had a bimodal rainfall distribution, with one peak occurring in April and May and the other in September and October. December and January typically received the lowest amounts of rainfall. The average yearly rainfall can vary from 1058 to 1453 mm. The mean monthly temperature fluctuates between 17 and 25 °C.

The Gidabo watershed comprised various land cover types such as agroforestry, farmland, shrubland and grassland, forest, settlement, and waterbodies. The main soil types in this watershed were chromic vertisols, dystric nitisols, eutric cambisols, pellic vertisols, eutric nitisols, orthic luvisols, and chromic luvisols. Geologically, this watershed was covered by flood basalt in the south–east, alluvial and lacustrine deposits in the south–west, volcanoclastic rocks in the north, rhyolitic volcanic in the center, and volcanic deposits and landforms in the northeast corner.

### 2.2 Data Collection

**Figure 2:**
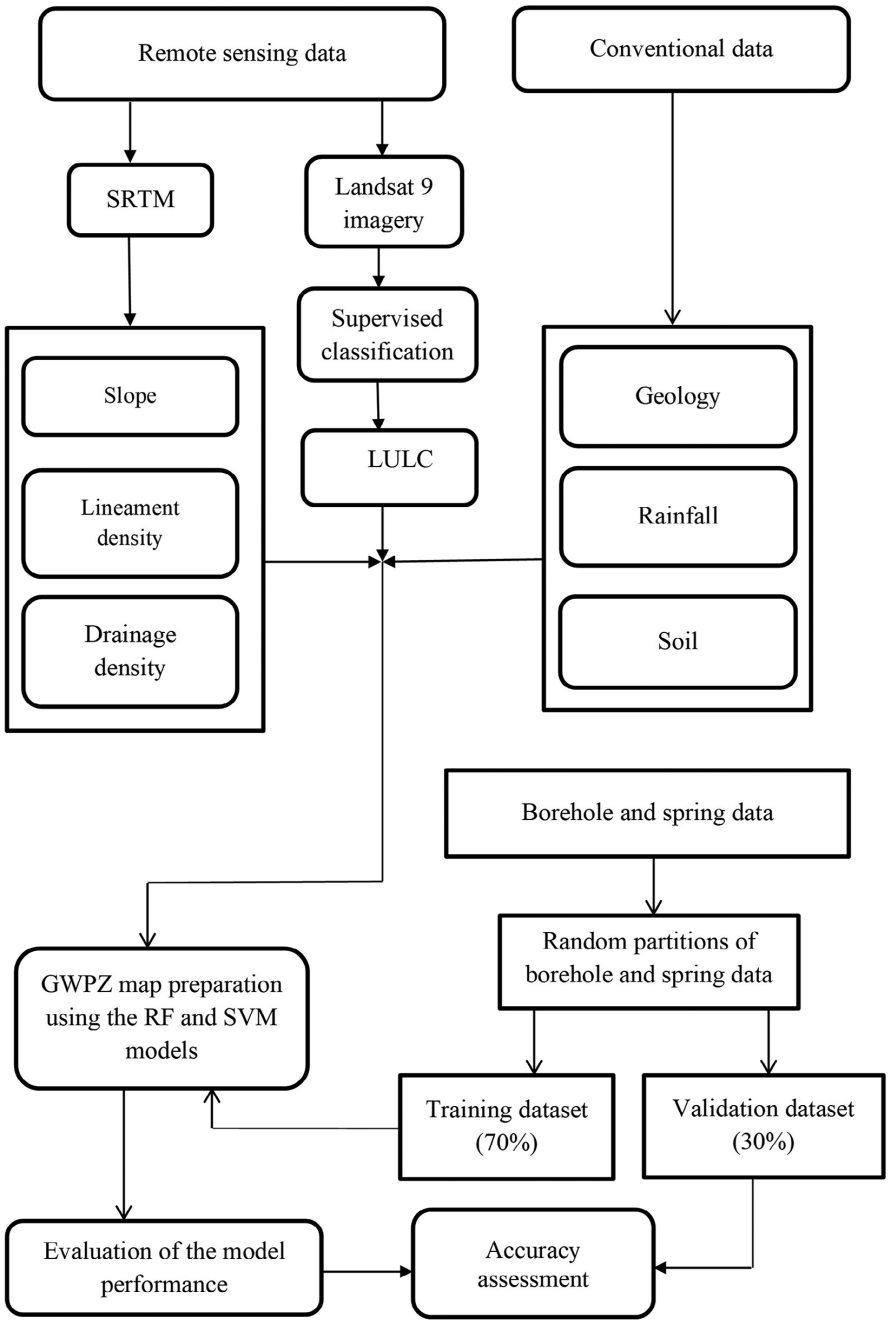
The methodology used for mapping GWPZs.

Mapping GWPZs with GIS‐based RF and SVM models involved gathering pertinent data from different sources, including the USGS Earth Explorer, the Ethiopian Meteorology Institute (EMI), and Ethiopia's Ministry of Water and Energy (MWE). **Table** **1** offers a comprehensive outline of the data sources used in this study. Following processing and integrating all relevant datasets into a single raster dataset in ArcGIS, the borehole and spring yield data were split into two sets: 70% for training and 30% for validation. The RF and SVM models were trained using the training data to generate GWPZs in the study area. Finally, the validation datasets were applied to evaluate the RF and SVM models' performance. **Figure** **2** depicts the methodology employed in this study to generate GWPZs.

**Table 1:** Data source summary.

Data	Source		Uses
SRTM DEM	https://earthexplorer.usgs.gov/	30 m × 30 m /2014	For creating maps of slope, lineament density, and drainage density
Soil type	MWE	Shapefile	For preparing a soil map
Geology map	MWE	Shapefile	For preparing a geology map
Landsat 9 imagery	https://earthexplorer.usgs.gov/	30 m × 30 m /2023	For generating a LULC map
Rainfall data	EMI	Daily/1993‐2022	To prepare a mean yearly rainfall map
Borehole and spring data	MWE	Yield [*Ls* ^−1^]/2014	To train and validate the RF and SVM models

SRTM = Shuttle Radar Topography Mission, DEM = Digital Elevation Model.

### 2.3 Thematic Layer Preparation

**Figure 3:**
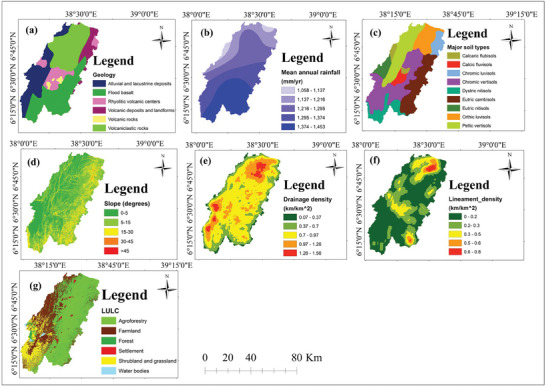
Thematic layers used to map GWPZs: a) geology, b) mean annual rainfall, c) soil, d) slope, e) drainage density, f) lineament density, and g) LULC.

A groundwater potential (GWP) map was generated by meticulously choosing and arranging thematic layers that impact the presence and movement of groundwater. The following factors influence GWPZs: geology, drainage density, rainfall, slope, LULC, lineament density, and soil.^[^ ^24^, ^25^, ^26^ ^]^ The GWPZs in the study area were identified using the seven thematic maps depicted in **Figure** **3**.

#### 2.3.1 Geology

The geological attributes of aquifers, including the composition of rocks, their thickness, structural features, and permeability, all significantly affect the presence and distribution of groundwater.^[^ ^27^ ^]^ Porosity determines a rock's ability to store water, whereas permeability determines its ability to convey water. Porosity affects how much water may be held in the geological substrate; however, permeability governs how the pore spaces are interrelated and transmit water.^[^ ^28^ ^]^ Geology has significance in the evaluation of GWP by influencing the hydraulic properties of rock, which directly affect water infiltration and percolation.^[^ ^29^ ^]^

According to geological data obtained by MWE and processed in ArcGIS 10.8, the study area encompasses the following geological formations: flood basalt covers 30.64%, volcanoclastic rocks cover 28.83%, alluvial and lacustrine deposits constitute 21.72%, rhyolitic volcanic centers make up 10.05%, volcanic deposits, and landforms account for 7.36%, and volcanic rocks cover 1.4% (Figure 3a).

#### 2.3.2 Rainfall

Groundwater was primarily derived from rainfall and replenished through infiltration and percolation processes. As a result, fluctuations in the water table within the aquifer were directly influenced by the quantity of rainfall that infiltrates into the subsurface.^[^ ^30^ ^]^ Rainwater is the main contributor to groundwater recharge, so it must be considered when mapping GWPZs.^[^ ^31^ ^]^ The average yearly rainfall in this study area was computed in ArcGIS with inverse distance weighted (IDW) interpolation techniques based on 30‐year rainfall data (1993‐2022) from six stations (Yergalem, Dilla, Billate, Hagere Selam, Haisawita, and Yirgachefe). Yearly rainfall was divided into five categories for simplicity of visualization and interpretation: 1058–1137 mm covers 3.07% of the study area, 1137–1216 mm covers 17.88%, 1216–1295 mm covers 27.37%, 1295–1374 mm covers 23.47%, and 1374–1453 mm covers 28.21% (Figure 3b).

#### 2.3.3 Soil

The soil type in an area significantly affects its infiltration rate and water‐holding capacity, playing a crucial role in determining GWPZs.^[^ ^32^, ^33^ ^]^ The soil type for this study area was provided by MWE, then projected to a suitable coordinate system and trimmed to match the study area's boundaries using ArcGIS 10.8. The study area comprised the following soil types: chromic vertisols cover 28.30%, eutric cambisols cover 18.57%, pellic vertisols cover 18.55%, orthic luvisols cover 10.75%, dystric nitisols cover 8.47%, chromic luvisols cover 5.13%, eutric nitisols cover 4.65%, calcic fluvisols cover 2.91%, and calcaric flubisols cover 2.68% of the study area (Figure 3c).

#### 2.3.4 Slope

The slope influenced how water seeps from the ground surface into the soil. The relationship between slope and soil infiltration rate demonstrated an inverse proportionality.^[^ ^34^ ^]^ A catchment's flow velocity increased with its slope, reducing infiltration and increasing surface runoff. Therefore, slope emerged as a significant factor influencing soil infiltration rate, thus impacting the delineation of GWPZs in a region.

Slopes in the geographical area fall into five categories: very gentle (0–5°), gentle (5–15°), moderate (15–30°), steep (30–45°), and very steep (>45°).^[^ ^35^ ^]^ The study area's slope map, generated with ArcGIS version 10.8 from the DEM, revealed that 39.55% consists of very gentle slopes, 44.81% gentle slopes, 14.31% moderate slopes, 1.28% steep slopes, and 0.05% very steep slopes (Figure 3d).

#### 2.3.5 Drainage Density

Drainage density can be defined as the aggregate length of channels per area of a watershed (Equation 1) and exhibits an inverse correlation with permeability and infiltration. Increased drainage density causes lower permeability, less infiltration, and more surface runoff. Thus, high drainage density suggests a low GWP for the area, which must be considered while mapping GWPZs.^[^ ^34^, ^36^ ^]^

(1) $$DD=\frac{L_{T}}{A}$$

where:

*DD =* drainage density [km.km^−2^],
 
*L*_*T*_ *=* overall channel length [km],
*A =* watershed area [Km^2^].

The map showing drainage density in the study area was created using ArcGIS version 10.8, with a DEM as the primary data source. The drainage density map depicts values ranging from 0 to 1.56 km.km^−2^ (Figure 3e). These are divided into five categories: 0.07–0.37 km.km^−2^ (very low), 0.37–0.7 km.km^−2^ (low), 0.7–0.97 km.km^−2^ (moderate), 0.97–1.26 km.km^−2^ (high), and 1.26–1.56 km.km^−2^ (very high). Regions with higher drainage density have a lower GWP.

#### 2.3.6 Lineament Density

Lineament density can be estimated by dividing the aggregate length of identified lineaments by the watershed area under examination, as illustrated in (Equation 2).^[^ ^37^ ^]^ Areas identified by a high density of lineaments show significant potential for groundwater resources.^[^ ^38^ ^]^ As a result, lineament density might be considered one of the important elements in defining GWPZs.

(2) $$Ld=\frac{\sum_{i=1}^{n}L_{i}}{A}$$

where:

*Ld* = lineament density [LL^−2^],
$\sum_{i=1}^{n}L_{i}$ *=* total length of lineaments [L],
*A =* the watershed's unit area [L^2^].

The map depicting lineament density in the investigated area was created from the DEM using ArcGIS version 10.8. According to the lineament density map (Figure 3f), its values vary from 0 to 0.8 km.km^−2^. These can be categorized into five groups: 0–0.2 km.km^−2^ (very low), 0.2–0.3 km.km^−2^ (low), 0.3–0.5 km.km^−2^ (moderate), 0.5–0.6 km.km^−2^ (high), and 0.6–0.8 km.km^−2^ (very high).

#### 2.3.7 Land Use and Land Cover (LULC)

LULC refers to identifying and characterizing how humans use and cover the Earth's surface. Groundwater was most prevalent in water bodies and wetlands, followed by forest cover, agriculture, and barren land and settlement areas.^[^ ^39^ ^]^ As a result, LULC was considered when defining GWPZs. Landsat imagery was sourced from the USGS Earth‐Explorer website and employed to generate a LULC map for the study area in ArcGIS, using supervised image classification techniques. The study area was predominantly encompassed by agroforestry, covering 48.18%, followed by farmland at 32.73%, with shrubland and grassland accounting for 14.26%, a forest at 3.01%, settlement areas at 1.28%, and water bodies at 0.53% (Figure 3g).

### 2.4 Borehole and Spring Inventory Map

**Figure 4:**
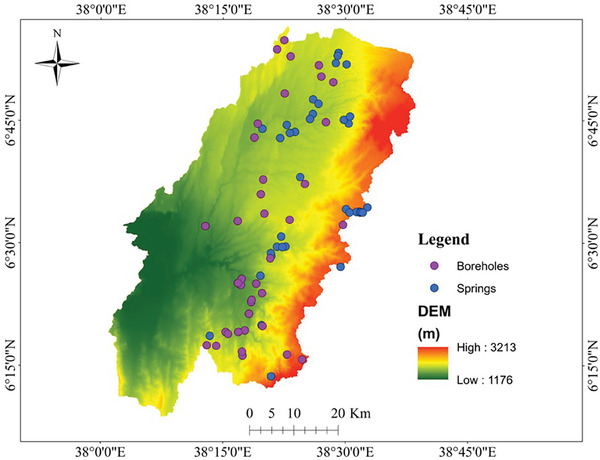
Map illustrating the locations of 39 boreholes and 36 springs in the study area.

A map depicting the precise locations of springs and boreholes is required for GWP investigations in a specific area. This map gives critical information about the spatial distribution of groundwater within the specified region. This study comprised 75 locations of boreholes and springs (potential sites), with yields varying from 0.2 *Ls* ^−1^ to 30 *Ls* ^−1^, as provided by the MWE (**Figure** **4**). Furthermore, using the high‐resolution Google Earth search engine and Landsat 9 imagery, 22 non‐potential groundwater sites (including bare lands and settlement areas) and 20 potential sites (containing water bodies) were identified. The data obtained was split into two sets, with 70% designated for training and 30% for validation.^[^ ^10^, ^40^ ^]^

### 2.5 Random Forest (RF) Model

The RF model, an ensemble ML technique, is used for classification and regression tasks. During the training phase, several decision trees were generated. In classification, the RF combines the majority votes from all decision trees, while in regression, it computes the mean or average of forecasts from each tree to get the final prediction. RF was highly favored in ML for several reasons: it was mitigated overfitted by aggregating predictions from multiple decision trees, enhanced overall accuracy by combining outputs from several trees, and offered better interpretability than other ML algorithms, like neural networks.^[^ ^41^, ^42^, ^43^, ^44^ ^]^

The RF model was extremely effective for mapping GWPZs due to its ensemble approach, which integrated estimates from multiple decision trees to get a final prediction. This technique reduced overfitting while improving prediction accuracy. It was additionally effective at handling vast, high‐dimensional datasets, making them ideal for data with many variables. It additionally offered variable importance scores, which assisted in identifying the most essential variables for predicting GWPZs.^[^ ^23^ ^]^

### 2.6 Support Vector Machine (SVM)

A support vector machine (SVM) is a supervised ML approach applied to address regression and classification problems. It uses a subset of training points known as support vectors to establish the decision function and predict outcomes. SVM is currently used for mapping GWPZs because of its effectiveness in handling complex classification tasks, adaptability, and ability to establish clear decision boundaries between subclasses.^[^ ^20^, ^45^, ^46^ ^]^

### 2.7 Training and Running RF and SVM Models in ArcGIS

To implement the RF and SVM ML models in ArcGIS version 10.8 for mapping GWPZs, the following steps were undertaken: Geospatial datasets influencing groundwater occurrence, including slope, soil type, geology, lineament density, rainfall, drainage density, and LULC, were collected. These datasets were merged into a single raster layer using ArcGIS, serving as input for the RF and SVM classifiers. 70% of the acquired ground truth data was employed to create training sample features. Each dataset was classified according to its yield potential: very high, high, moderate, low, and very low. The RF and SVM models were run with the combined raster layer and training sample features as inputs. The RF model was trained with the recommended default values, which included 50 trees, a maximum tree depth of 30, and a maximum of 1000 samples per class, while the SVM model was trained with the recommended default value of 500 samples per class.

The results from executing the RF and SVM models provide a cross‐validation rate. Furthermore, the RF model offers variable importance. The models were trained to understand connections between input parameters and GWP classes. After training, the RF and SVM models were applied to the entire study area. The models analyze geospatial datasets from the study area and categorize them into specified GWP classes: very high, high, moderate, low, and very low. Finally, the RF and SVM models yielded a categorized raster map depicting the GWPZs in the study area.

When mapping GWPZs using the RF model, variable importance refers to the significance of different input variables in predicting GWP. Higher importance values indicate that the associated variables play greater roles in determining GWP. RF model commonly uses techniques including k‐fold cross‐validation to measure their performance during training. This technique partitions the dataset into k subgroups. The model undergoes training for k times, with every iteration using a new group of k‐1 subgroups for training and one subgroup reserved for validation. This technique ensures the model's performance is thoroughly tested across diverse data subgroups. A high cross‐validation rate indicates that the model is efficient and capable of generalizing to new, previously unknown data.^[^ ^47^ ^]^

Model tuning and k‐fold validation, commonly employed in other ML models, considerably improve the SVM model's performance. Model tuning entails altering the model's parameters to increase its efficiency. K‐fold validation gives a reliable evaluation by dividing the dataset into k subsections. The model is trained k times in each iteration, with a unique combination of k‐1 subsections for training and one subsection for validation. A high cross‐validation rate suggests the model is reliable and may be applied to new, previously unseen data.^[^ ^48^ ^]^

### 2.8 Evaluating Model Performance

The GWPZ classifications generated by the models were compared to known reference data (ground truth), which comprised 30% of the dataset and was reserved for validation. The performance of the GWPZ classification was evaluated in ArcGIS by creating a confusion matrix with random accuracy assessment points based on the size of the validation dataset and calculating the Kappa coefficient, overall accuracy, and Area Under the Receiver Operating Characteristic (ROC) curve. Implementing a confusion matrix yields the producer's accuracy (PA) and the user's accuracy (UA) for each class, as well as the overall accuracy (OA) and Kappa coefficient (*k*). It additionally reveals the true positives (TP), false positives (FP), true negatives (TN), and false negatives (FN). The OA^[^ ^49^ ^]^ and *k* ^[^ ^50^ ^]^ are defined by Equations (4) and (5), respectively.

Instances correctly classified based on the reference data are shown on the diagonal of the matrix, while misclassified are depicted by off‐ diagonal entries. PA calculates classification accuracy for each category by comparing it to the total instances in the corresponding column. UA evaluates classification accuracy for each category to the total cases in that row.

(3) $$\begin{array}{cc} \mathrm{OA} & =\frac{\mathrm{Number}\;\mathrm{of}\;\mathrm{instances}\;\mathrm{that}\;\mathrm{are}\;\mathrm{properly}\;\mathrm{classified}\;}{\mathrm{total}\;\mathrm{count}\;\mathrm{of}\;\mathrm{the}\;\mathrm{reference}\;\mathrm{instances}}\\ & \;*100\% \end{array}$$

(4) $$k=\frac{p_{o}-p_{e}}{1-p_{e}}$$

where:

 
*p_o_* = observed agreement,
 
*p_e_* = expected agreement.

The kappa coefficient spans from ‐1 to 1. A value close to 1 implies a strong agreement between the expected and observed categories. A value close to zero, on the other hand, means that the prediction is no better than what could be expected at random. A number close to −1 indicates a considerable divergence between the predicted and observed categories.^[^ ^50^ ^]^ A ROC curve is a graph that shows the performance of a classification model at all classification levels. It is generated by plotting the True Positive Rate (TPR) or sensitivity on the *y*‐axis and the False Positive Rate (FPR) or (1 − specificity) on the x‐axis.^[^ ^51^ ^]^ The area under the ROC curve (AUC) was computed using OriginLab, a data analysis software.

The area under the ROC curve (AUC) is a scalar number that quantifies the model's overall performance. It varies between 0 and 1, and a higher AUC suggests a more effective model. The AUC scale can be understood as follows: 0.9–1.0 represents excellent performance, 0.8–0.9 is very good, 0.7–0.8 is good, 0.6–0.7 is satisfactory, and 0.5–0.6 is unsatisfactory.^[^ ^52^ ^]^

(5) $$\mathrm{TPR}\;=\frac{\mathrm{TP}}{\mathrm{TP}+\mathrm{FN}}$$

(6) $$\mathrm{FPR}\;=\frac{\mathrm{FP}}{\mathrm{FP}+\mathrm{TN}}$$

## 3 Results

### 3.1 Variable Importance and Cross‐Validation Rate

**Figure 5:**
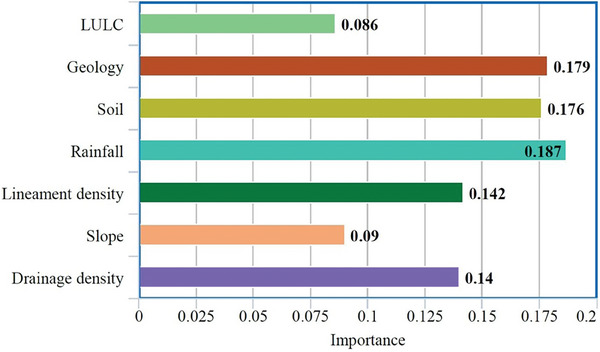
Map illustrating the variable importance outcomes derived from the RF model.

Running the RF model provided two important pieces of information: variable importance and cross‐validation rate and running the SVM model provided a cross‐validation rate. The variable importance results obtained from the RF model, aimed at predicting GWP within the study area, reveal the following contributions, listed in descending order of significance: Rainfall holds the highest importance at 0.187, followed by geology at 0.179, the soil at 0.176, lineament density at 0.142, drainage density at 0.140, the slope at 0.090, and LULC at 0.086 (**Figure** **5**). The cross‐validation rate for the trained RF model was 0.983, while the SVM model was 0.980.

### 3.2 Prediction Results of GWPZs

**Figure 6:**
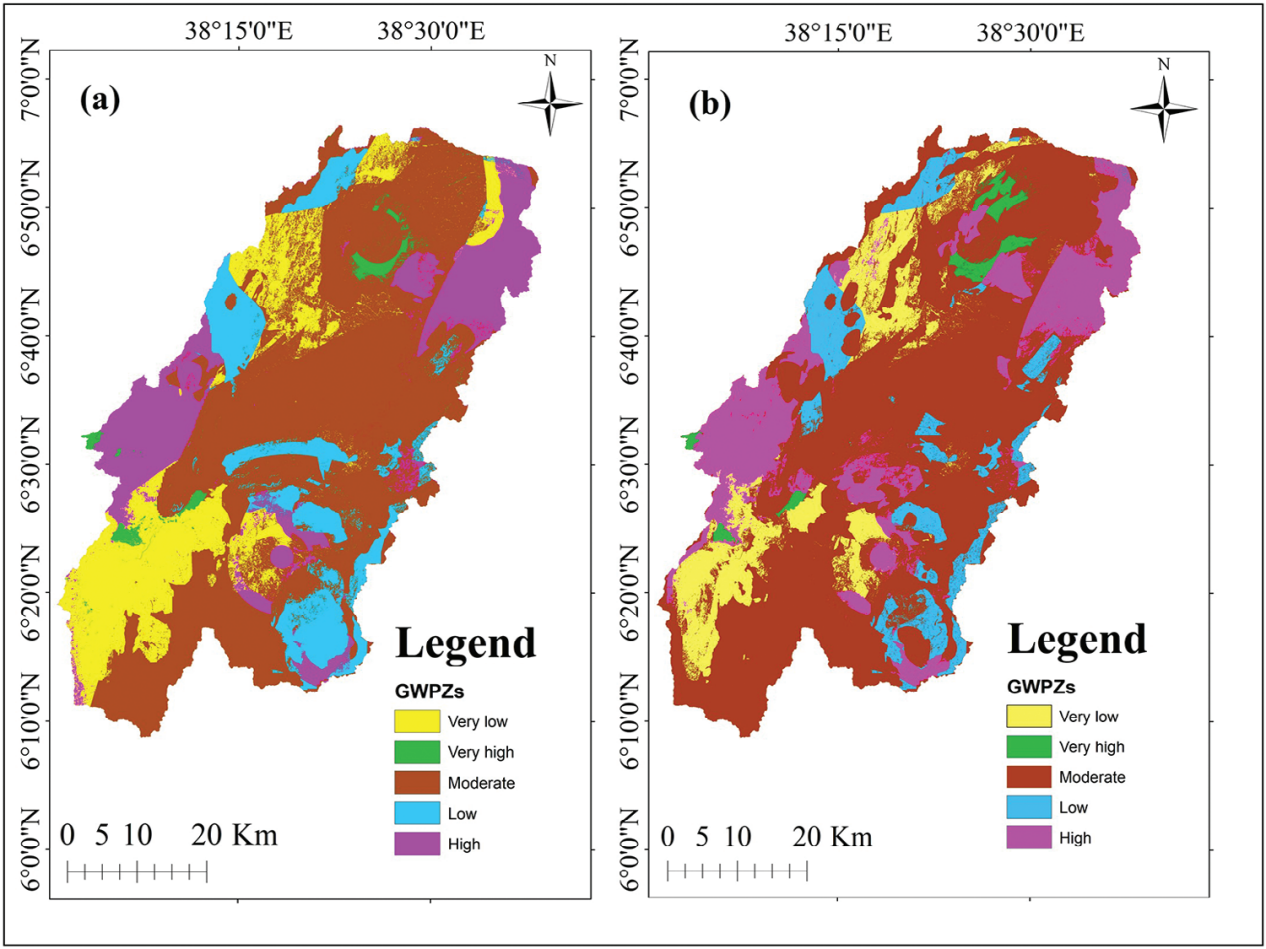
Map depicting GWPZs in the study area: a) RF model, b) SVM model.

The RF and SVM models generated raster maps depicting the GWPZs across the study area. The zones were divided into five categories: very low (17.28%), low (11.17%), moderate (54.57%), high (15.71%), and very high (1.27%) for the RF model (**Figure** **6**a), and very low (10.95%), low (8.34%), moderate (62.08%), high (16.89%), and very high (1.74%) for the SVM model (Figure 6b).

### 3.3 Evaluation of Model Performance Results

**Figure 7:**
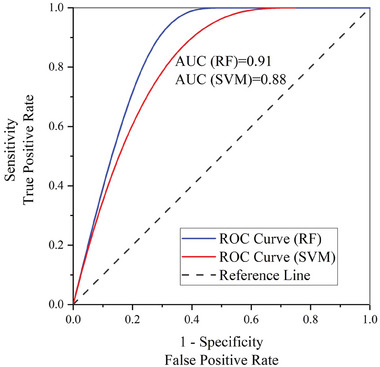
ROC curves and AUC.

After mapping the GWPZs in the study area using the RF model, the evaluation revealed an overall accuracy of 0.95 and a Kappa coefficient of 0.91 (**Table** **2**). Likewise, mapping the GWPZs using the SVM model revealed an overall accuracy of 0.92 and a Kappa coefficient of 0.87 (**Table** **3**). The area under the ROC curve for the RF model was 0.91, while the SVM model was 0.88 (**Figure** **7**).

**Table 2:** Output of confusion matrix for the RF model.

OID	Class_Value	C_1	C_2	C_3	C_4	C_5	Total	U_Accuracy	Kappa
0	C_1	7	0	0	0	0	7	1	0
1	C_2	0	6	0	0	0	6	1	0
2	C_3	0	2	19	0	0	21	0.90	0
3	C_4	0	0	0	2	0	2	1	0
4	C_5	0	0	0	0	1	1	1	0
5	Total	7	8	19	2	1	37	0	0
6	P_Accuracy	1	0.75	1	1	1	0	0.95	0
7	Kappa	0	0	0	0	0	0	0	0.91

C_1 = very low, C_2 = low, C_3 = moderate, C_4 = high, and C_5 = very high.

**Table 3:** Output of confusion matrix for the SVM model.

OID	Class_Value	C_1	C_2	C_3	C_4	C_5	Total	U_Accuracy	Kappa
0	C_1	7	0	0	0	0	7	1	0
1	C_2	0	6	0	0	0	6	1	0
2	C_3	0	3	18	0	0	21	0.86	0
3	C_4	0	0	0	2	0	2	1	0
4	C_5	0	0	0	0	1	1	1	0
5	Total	7	9	18	2	1	37	0	0
6	P_Accuracy	1	0.67	1	1	1	0	0.92	0
7	Kappa	0	0	0	0	0	0	0	0.87

C_1 = very low, C_2 = low, C_3 = moderate, C_4 = high, and C_5 = very high.

## 4 Discussion

### 4.1 Addressing Water Scarcity: The Significance of GWPZ Mapping in the Gidabo Watershed

Communities in the Gidabo watershed experience water scarcity for domestic usage, especially during the dry season.^[^ ^6^ ^]^ Agriculture is the main land use in this area, which is fully dependent on rainfall, making it more susceptible to seasonal variations. Furthermore, increasing population and urbanization put additional strain on already limited water resources.^[^ ^7^ ^]^ Thus, evaluating GWP is vital for boosting agricultural output and meeting domestic water needs. Consequently, GWPZ mapping and evaluation are crucial for sustainable water management in any region.^[^ ^10^, ^53^ ^]^

As more high‐quality training data becomes readily accessible, ML techniques will gain effectiveness across various geographic regions and environmental situations. Access to high‐quality data enables ML models to perform better with more detailed and relevant information. This allows models to learn from a larger sample, resulting in accurate and reliable predictions that capture the complex nature of many situations. As a result, ML techniques can be tuned to specific applications, increasing their adaptability and effectiveness across geographical areas.^[^ ^54^ ^]^ As a result, quality data is available for training ML models, enabling them to effectively address the challenge of identifying GWPZs across various geographic regions.^[^ ^55^ ^]^ This study used two GIS‐based ML models, RF and SVM, to map GWPZs in the Gidabo watershed.

### 4.2 Insights from RF and SVM Models: Variable Importance and Cross‐Validation Rates for GWPZ Prediction

Figure 5 depicts the RF model's variable importance results, which reveals a different hierarchy of factors influencing GWP in the study area. Rainfall was identified as the most critical variable, with an importance score of 0.187. This high relevance highlights precipitation's vital part in replenishing groundwater resources, as it influences the quantity of water available for penetration into the aquifer.^[^ ^30^ ^]^ Geology followed closely with an important score of 0.179, indicating that the composition and structure of rock formations have significant effects on groundwater storage and movement.^[^ ^27^ ^]^ The soil's importance score of 0.176 underlines its role in controlling water infiltration and retention, which governs groundwater recharge rates.^[^ ^32^ ^]^

Lineament density, with a score of 0.142, indicates the presence of fractures and faults in geological formations, which can improve groundwater flow by creating channels for water movement.^[^ ^38^ ^]^ Drainage density, which has a score of 0.140, affects surface water distribution, infiltration rates, and runoff, ultimately influencing the quantity of water that recharges groundwater.^[^ ^34^ ^]^ Slope, with an importance score of 0.090, governs the rate at which water travels across the land surface, influencing infiltration rates and, thus, groundwater systems.^[^ ^34^ ^]^ LULC has a 0.086 importance score, suggesting that it influences the rate at which water replenishes the groundwater system.^[^ ^39^ ^]^

The cross‐validation rates indicate that the RF and SVM models performed quite well in predicting GWPZs, with the RF model achieving a slightly higher rate of 0.983 compared to 0.980 for the SVM model. This slight difference shows that, while both models are highly successful, the RF model may have a slightly higher predicted accuracy for this particular purpose. Generally, a high cross‐validation rate indicates that the models have great prediction capability and will likely apply successfully to new data.^[^ ^56^ ^]^

### 4.3 Groundwater Potential Zoning

Figure 6 depicts the GWPZs identified using the RF and SVM models. The results show that areas with very high GWP (highlighted in green) primarily have water bodies, dense natural springs, volcanoclastic rock formations, and gentle slopes. This demonstrates the important influence of water bodies on groundwater occurrence.^[^ ^39^ ^]^ Furthermore, it suggests that areas with gentle slopes have much higher GWP due to reduced runoff and increased infiltration rates.^[^ ^57^ ^]^ Additionally, areas dominated by volcanoclastic rocks are known for having significant groundwater reserves due to their high porosity and permeability.^[^ ^58^ ^]^

High GWPZs (pink color), situated in the southwest corner of the study area, are primarily accompanied by gentle slopes and forest cover. Likewise, in the northeast corner, similar zones occur in places with chromic luvisols, volcanic deposits, and landforms. This emphasizes the importance of forests in groundwater occurrence because they act as natural sponges, absorbing and retarding the movement of water. It helps to reduce surface runoff and improve infiltration.^[^ ^39^ ^]^ Furthermore, areas with gentle slopes tend to demonstrate high GWP via encouraging infiltration and lowering surface runoff.^[^ ^57^ ^]^

The chromic luvisols are also characterized by moderately well‐drained, medium‐textured soils, particularly silt loam.^[^ ^59^ ^]^ Silt loam soils possess a good water‐holding capacity, indicating their potential to store and transmit groundwater.^[^ ^60^ ^]^ Furthermore, volcanic deposits and landforms often have substantial groundwater reserves due to their high porosity and permeability caused by their vesicular, fractured, and fissured nature.^[^ ^61^ ^]^

Conversely, the bottom southwest corner has very low GWPZs (yellow color), primarily found in shrubland and grassland areas. These regions often have sparse vegetation cover, and their soils exhibit lower organic matter content and less developed structures than forested areas. This indicates a limited capacity for water retention and groundwater recharge, suggesting a decrease in groundwater availability.^[^ ^62^ ^]^

### 4.4 Model Accuracy and Performance Metrics for Groundwater Potential Zonation: RF Versus SVM

The evaluation of the GWPZs maps generated by the RF and SVM models demonstrates that both models perform successfully. The RF model achieved an overall accuracy of 0.95 and a Kappa coefficient of 0.91, indicating high accuracy and agreement between predicted and observed GWPZs (Table 2). Similarly, the SVM model performed effectively, with an overall accuracy of 0.92 and a Kappa coefficient of 0.87, although slightly less than the RF (Table 3). These findings show that both models are competent in identifying GWPZs, but the RF model surpasses the SVM model slightly. The area under the ROC curve (AUC) for the RF model was 0.91, whereas the SVM model achieved an AUC of 0.88 (Figure 7). This suggests that the RF model has a slightly greater ability to identify distinct GWPZs, as a higher AUC implies better performance in class differentiation. While both models have good predictive power, the RF model has a greater AUC, indicating that it might give a more dependable estimate of GWPZs over the study area. Generally, the results reveal that the RF model outperforms the SVM model, despite both having reasonable accuracy.^[^ ^21^, ^63^ ^]^

### 4.5 Scope and Limitation of the Models

GIS‐based GWPZ mapping with RF and SVM models gives valuable information by analyzing important conditioning factors such as geology, soil, slope, etc. These models excel at identifying probable GWPZs or productive aquifer zones (underground layers that may yield considerable amounts of water for extraction via wells or boreholes) and providing guidance for targeted test drilling. However, the study has some limitations, particularly its reliance on generalized conditioning factors that lack consideration for specific aquifer properties such as porosity (water storage ability) and permeability (water transmission ability), which have significance when assessing aquifer productivity.^[^ ^64^ ^]^

To address these constraints, future investigations should increase the training dataset by including more boreholes and springs, this can enhance model accuracy. Furthermore, a solid understanding of the aquifer's unique properties, particularly porosity and permeability, is essential. Future studies should focus on incorporating these vital aquifer properties into ML techniques and GIS to improve the realism and practicality of GWPZ mapping, eventually leading to more effective water resource management strategies.

## 5 Conclusion

Machine learning (ML) models are gaining prominence as a viable method for groundwater management. Its ability to recognize complex relationships across large datasets and make reliable predictions opens novel opportunities for investigating GWPZs and offers useful information for borehole drilling initiatives. This study utilized GIS‐based RF and SVM ML models to evaluate GWPZs in the Gidabo watershed. This was achieved by utilizing seven thematic maps that impact groundwater occurrences, including drainage density, LULC, lineament density, rainfall, geology, soil type, and slope. Furthermore, borehole and spring yields, alongside non‐potential sites, were used to train and validate the performance of RF and SVM models. The results reveal that both models effectively classified GWPZs into five categories: very low, low, moderate, high, and very high. The RF model achieved an AUC of 0.91, compared to 0.88 for the SVM model. Both models are competent at identifying GWPZs, however the RF model performs slightly better.

The classified GWPZ map reveals that high GWPZs are typically situated in areas with water bodies, natural springs, gentle slopes, and forest cover. In contrast, low GWP zones are mostly found in shrubland and grassland areas. This study will significantly enhance the selection of ideal drilling sites for water wells, thereby meeting the community's water needs within the watershed. It will assist farmers in selecting suitable crops and irrigation systems, thus improving agricultural output and water management. It also provides vital information for decision‐makers concerning groundwater extraction, allocation, and sustainable usage, ensuring long‐term water resource management and strategic planning.

## Author Contributions

M.M.M. gathered data from multiple sources, analyzed and interpreted it, and contributed to the paper's writing. T.K.L. Gathered data from multiple sources, analyzed and interpreted it, and contributed to the paper's writing. A.A.E. gathered data from multiple sources, analyzed and interpreted it, and contributed to the paper's writing.

## Conflict of Interest

The authors declare no conflict of interest.

## Supporting information

Supporting Information

## Data Availability

The data that support the findings of this study are available from the corresponding author upon reasonable request.
