# Supplementary material for: Evaluation of Groundwater Potential Zones Using GIS‐Based Machine Learning Ensemble Models in the Gidabo Watershed, Ethiopia
Source: Glob Chall. 2024 Oct 16;8(12):2400137. doi: 10.1002/gch2.202400137 (PMC11637779; doi:10.1002/gch2.202400137)
Supplement: Supplementary file 1 — Supporting Information [file GCH2-8-2400137-s001.pdf]

Global
Challenges
Open Access

Supporting Information

for *Global Challenges.*, DOI 10.1002/gch2.202400137

Evaluation of Groundwater Potential Zones Using GIS-Based Machine Learning Ensemble Models in the Gidabo Watershed, Ethiopia

Mussa Muhaba Mussa, Tarun Kumar Lohani and Abunu Atlabachew Eshete*

	**Boreholes**	X UTM [m]	Y UTM [m]	Elevation [m]	SWL [m]	Q [Ls^{-1}]	Rate
1	Felka	413096	722360	1438	94.7	4.4	Moderate
2	Alawo orke	429316	762264	1963	94.7	4.4	Moderate
3	Qorke	426144	732862	1786	22.3	4.4	Moderate
4	Tara Mesa	438753	758658	1829	7.1	2	Moderate
5	Dila Town	421122	709070	1455	24.05	7.8	Moderate
6	Qosoricha	426437	725183	1849	56.59	5.8	Moderate
7	Debeqa	420400	723500	1645	55.3	5	Moderate
8	Dese	424178	742349	1621	0.5	6.5	Moderate
9	Suqo	434956	692250	2844	5.4	0.3	Low
10	Wirama	444196	722657	2705	12.4	0.2	Low
11	Dabota	421475	693132	1792		3	Moderate
12	Wenago Town	417650	698467	1752		2	Moderate
13	Mechiso	421297	710450	1451	47.5	7.7	High
14	Sika	431539	693360	2588	30.1	0.7	Low
15	Alawo Ano	432358	760688	1833	50.6	4.3	Moderate
16	Amba	425996	699819	1994	40.55	2.7	Moderate
17	Oda Mike	420446	709514	1440	28.5	7.1	High
18	Adola	424562	709368	1575	0.5	20	Very high
19	Wetiko	421380	694051	1759		2	Moderate
20	Gosa	425904	707230	1724		3	Moderate
21	Kera Sodity	413406	695488	1937		3	Moderate
22	Jemjemo	415535	695342	2017		2	Moderate
23	Chichu	422937	702591	1556		2	Moderate
24	Kebado Town	427747	715067	1819	11.5	5	Moderate
25	Shoicha	435581	731867	1906	85.16	5	Moderate
26	Telamo	441999	754828	1872	16	1	Moderate
27	Sedeqa	439302	756099	1837	29	2	Moderate
28	Sadamo dikicha	430942	764320	2026	42	1.5	Moderate
29	Haseharo	421977	698810	1653	12.4	1	Moderate
30	Semen Mesenkela	431035	752301	1742	9.75	6.6	Moderate
31	Finchweha	418187	698025	1752		13.5	High
32	Tumata Cheracha	420517	698489	1658		12.5	High
33	Hole Kena	440336	745829	1836		12	High

34	Chechu-1	423417	705225	1525		10	High
35	Chechu-2	423544	705692	1528		10	High
36	Halo	432143	723778	1972		4.2	Moderate
37	Cheko Fara	425634	729575	1846		5	Moderate
38	Legedura River	424562	709376	1583		13.4	High
39	Yirga Alem	424916	745491	1631	7.8	6	Moderate

SWL=Static Water Level, Q = discharge rate=pump yield, UTM= Universal Transverse Mercator

	**Springs**	X UTM[m]	Y UTM[m]	Elevation [m]	Q $[Ls^{-1}]$	Rate
1	Adola	425533	711155	1623	0.2	Low
2	Shacho	429258	717705	1764	0.25	Low
3	Kebado Town	428002	715587	1868	0.33	Low
4	Shacho	431190	717750	1829	1	Moderate
5	Shacho	430472	717632	1794	2	Moderate
6	Setamo	427895	716252	1776	2.5	Moderate
7	Tumiticha	425762	700021	1985	3.79	Moderate
8	Dekuwa Hashere	427942	688447	1882	15	High
9	Abera Atila	443635	713162	2765	2	Moderate
10	Lelahoncho	430214	720023	1863	0.58	Low
11	Gidibo	434521	733422	1697	4.7	Moderate
12	Chancho	425974	744340	1679	12	High
13	Dobe Toga	445005	758861	1961	1	Moderate
14	Awada	433367	743629	1788	1.2	Moderate
15	Shoye	437386	747676	1720	1.5	Moderate
16	Wene Nata	429986	742222	1729	2	Moderate
17	Galuko Haro	443192	761468	2035	5	Moderate
18	Huletenya Fero	445479	745514	1957	2	Moderate
19	Teticha	447847	725629	1937	6	Moderate
20	Teticha	447241	725545	2679	2	Moderate
21	Teticha	448078	725432	2699	1.4	Moderate
22	Bokaso Town	444376	746350	1938	1.2	Moderate
23	Teticha	444877	726185	2500	1	Moderate

24	Teticha	448303	725501	2665	1	Moderate
25	Oudessa	449726	726593	2680	1	Moderate
26	Teticha	448656	725428	2673	1.2	Moderate
27	Sodity	414069	697595	1877	1.2	Moderate
28	Abada	432180	743397	1684	10	High
29	Cowana	442590	759154	1898	3	Moderate
30	Moto	437441	750968	1766	3	Moderate
31	Saint Mary	436732	746512	1717	30	Very high
32	Yoyota Gasha	445796	747113	1988	1.8	Moderate
33	Meleya	445667	725439	2619	1.5	Moderate
34	Garden	443000	760814	1944	6.1	Moderate
35	Meka Catolic	438663	749981	1769	2.16	Moderate
36	Ras Desta Ber	431484	745197	1686	5	Moderate

Q= spring yield, UTM= Universal Transverse Mercator

Additional Control Points from Landsat 9 image and Google Earth

	Code	X UTM [m]	Y UTM[m]	Rate
1	VH1	399726	708347	Very high
2	VH2	400658	708474	Very high
3	VH3	410098	712850	Very high
4	VH4	411050	713962	Very high
5	VH5	395825	721200	Very high
6	VH6	396102	721915	Very high
7	H1	398509	722600	High
8	H2	399197	723103	High
9	H3	399779	720245	High
10	H4	400097	720642	High
11	H5	403123	719277	High
12	H6	403335	719690	High
13	H7	446433	745909	High
14	H8	446228	745966	High
15	H9	448760	744684	High
16	H10	448633	744369	High
17	H11	457239	744725	High
18	H12	457445	744507	High
19	H13	457678	747574	High
20	H14	457811	747606	High

21	L1	417077	730939	Low
22	L2	417596	731324	Low
23	L3	414455	738618	Low
24	L4	414646	738968	Low
25	L5	430203	756795	Low
26	L6	430362	757033	Low
27	L7	432537	762447	Low
28	L8	432640	762860	Low
29	VL3	438283	759464	Very low
30	VL4	438316	759960	Very low
31	VL5	422491	744416	Very low
32	VL6	423532	746351	Very low
33	VL7	426542	738843	Very low
34	VL8	427071	740183	Very low
35	VL9	430246	737768	Very low
36	VL10	430891	738810	Very low
37	VL11	423069	708995	Very low
38	VL12	423681	708284	Very low
39	VL13	420854	702281	Very low
40	VL14	421647	702992	Very low
41	VL15	411416	710877	Very low
42	VL16	415205	71168	Very low

30 years' mean annual rainfall

	Name	X UTM[m]	Y UTM [m]	Elevation [m]	Rainfall [mm]
1	Billate	399202.326	754191.522	1361	820.85
2	Dilla	423352.919	705323.872	1515	1453.07
3	Hagere Selam	447449.336	717180.46	2809	1358.92
4	Haisawita	451575.172	762931.397	2267	1123.57
5	Yergalem	437261.083	751737.926	1786	1255.70
6	Yirga Chefe	412377.743	680004.375	1856	1409.02

UTM= Universal Transverse Mercator

Gidabo Watershed, Ethiopia

Sunday, August 11, 2024
